# Modeling Alzheimer’s disease: from past to future

**DOI:** 10.3389/fphar.2013.00077

**Published:** 2013-06-19

**Authors:** Claudia Saraceno, Stefano Musardo, Elena Marcello, Silvia Pelucchi, Monica Di Luca

**Affiliations:** ^1^Dipartimento di Scienze Farmacologiche e Biomolecolari, Università degli Studi di MilanoMilano, Italy; ^2^Centre of Excellence on Neurodegenerative Diseases, Università degli Studi di MilanoMilano, Italy

**Keywords:** Alzheimer’s disease, animal models, computational model, secretases, amyloid

## Abstract

Alzheimer’s disease (AD) is emerging as the most prevalent and socially disruptive illness of aging populations, as more people live long enough to become affected. Although AD is placing a considerable and increasing burden on society, it represents the largest unmet medical need in neurology, because current drugs improve symptoms, but do not have profound disease-modifying effects. Although AD pathogenesis is multifaceted and difficult to pinpoint, genetic and cell biological studies led to the amyloid hypothesis, which posits that amyloid β (Aβ) plays a pivotal role in AD pathogenesis. Amyloid precursor protein (APP), as well as β- and γ-secretases are the principal players involved in Aβ production, while α-secretase cleavage on APP prevents Aβ deposition. The association of early onset familial AD with mutations in the APP and γ-secretase components provided a potential tool of generating animal models of the disease. However, a model that recapitulates all the aspects of AD has not yet been produced. Here, we face the problem of modeling AD pathology describing several models, which have played a major role in defining critical disease-related mechanisms and in exploring novel potential therapeutic approaches. In particular, we will provide an extensive overview on the distinct features and pros and contras of different AD models, ranging from invertebrate to rodent models and finally dealing with computational models and induced pluripotent stem cells.

## INTRODUCTION

Alzheimer’s disease (AD), the major cause of dementia, is associated with progressive memory loss and severe cognitive decline. Initially mild cognitive impairment and deficits in short-term and spatial memory appear, but the symptoms become more severe with disease progression, eventually culminating in loss of executive function.

The immense social and economic ramifications of AD have generated major efforts toward obtaining a better understanding of the disease and toward developing therapeutic agents for its treatment. Regrettably, however, there is no remission in the progression of AD, nor are there any disease-stabilizing drugs currently available.

The neuropathology is characterized by the presence of intracellular and extracellular protein or peptide aggregates: the hyperphosphorylated tau, assembled into the paired helical filaments within neurofibrillary tangles (NFTs) and swollen neuritis (Grundke-Iqbal et al., 1986), and the Amyloid β (Aβ) peptides existing in extracellular β-pleated sheet conformations assembled into oligomers, in amyloid plaques (Glenner and Wong, 1984).

Although AD pathogenesis is multifaceted and difficult to pinpoint, genetic and pathological evidence strongly supports the amyloid cascade hypothesis of AD, which posits that Aβ has an early and vital role in AD, since it triggers a cascade of events leading to synaptic dysfunction, tau pathology, and neuronal loss (Hardy and Selkoe, 2002).

The production of Aβ is mediated by the concerted action of two different secretases, namely β-secretase [β-site amyloid precursor protein (APP)-cleaving enzyme, BACE] and γ-secretase, showing a proteolytic action on the APP (Haass, 2004). BACE cleaves at the N-terminus of the Aβ sequence, releasing into the extracellular space a soluble fragment, designated sAPPβ, and leaving attached to the cellular membrane a 99 aminoacids (aa) long C-terminal fragment (CTF99), which is then cleaved by γ-secretase at the C-terminus of the Aβ domain (**Figure 1**). Proteolysis by γ-secretase is heterogeneous: most of the full-length Aβ species produced is a 40-residues peptide (Aβ40), whereas a small proportion is a 42-residues COOH-terminal variant (Aβ42; Esler and Wolfe, 2001). γ-secretase is a multimeric complex thought to be made up of an essential quartet of transmembrane proteins – presenilin 1 (or 2), nicastrin, Aph-1 (anterior pharynx-defective 1), and Pen-2 (presenilin enhancer 2; Edbauer et al., 2003).

**FIGURE 1:**
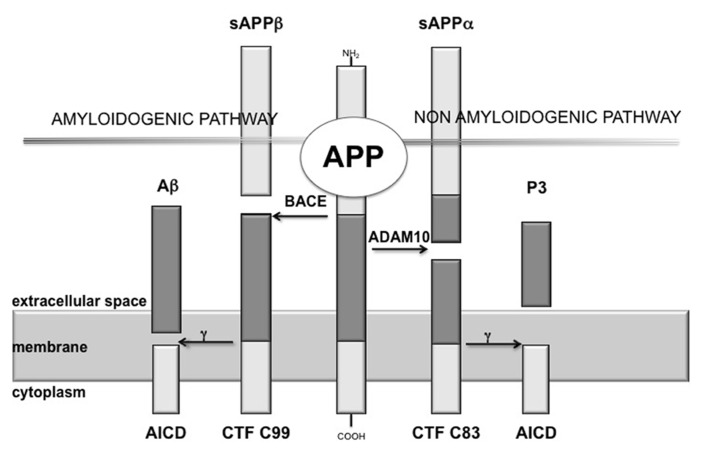
**Scheme of amyloid cascade.** APP is the precursor of the amyloid β peptide (Aβ), here it is shown the proteolytic cleavage of APP by the α-secretase (ADAM10), which precludes the formation of amyloidogenic peptides and leads to the release of sAPPα, while β-secretase (BACE) cleaves APP at the N-terminus of the Aβ sequence, releasing into the extracellular space sAPPβ. Proteolysis of the APP C-terminal fragments by γ-secretase is the last processing step resulting in the generation of p3 and Aβ, respectively.

On the other hand, α-secretase is the main protagonist of the physiological APP metabolic pathway.

It has been demonstrated that the constitutively cleaving α-secretase activity is selectively mediated by ADAM10 (a disintegrin and metalloprotease) in neurons (Jorissen et al., 2010; Kuhn et al., 2010). ADAM10 cleaves APP on the C-terminal side of residue 16 of the Aβ sequence, destroying this amyloidogenic component and, thus, preventing the formation and deposition of plaques (**Figure 1**).

Two different forms of AD have been described: (i) early onset familial AD (FAD; Hornsten et al., 2007), associated with mutations in the genes encoding APP, presenilin 1 (*PSEN1*) and presenilin 2 (*PSEN2*), and accounting for less than 5% of total AD; and (ii) a more common late onset AD (LOAD), in which a main risk factor is expression of the ε4 allele of the apolipoprotein E gene (*APOE*). Specifically, the presence of two ε4 alleles increases by approximately 12-fold the risk for AD and lowers the age of onset of the disease by about 15 years (Kim et al., 2009).

Alzheimer’s disease-causing mutations in APP result in an overall increase of the production of Aβ. More than 60 mutations have been identified in presenilin 1 and they affect APP processing so that more Aβ 42 is produced (Selkoe, 2000). Mutations in presenilin 2 are rare but are also thought to affect the processing of APP toward amyloidosis. Mutations in the gene encoding tau (*MAPT*) have not been linked to AD, although they are associated with other dementias such as frontal temporal lobe dementia (Goedert and Spillantini, 2000).

The links between APP, presenilin 1, and FAD provided a potential tool of generating animal models of the disease to turn these genetic information, in the form of pathogenic mutations, into clinically useful drugs against the human disease.

Animal models of AD have been designed to reproduce various components of the pathological, biochemical, and behavioral characteristics of AD in order to understand the consequences of the pathological and biochemical changes that occur as the disease progresses, and investigate the effectiveness of potential pharmacotherapies (Wenk and Olton, 1987, 1989). An animal model is useful only if it leads to the development of an effective therapy or provides a better understanding of the biological mechanisms that underlie the symptoms of the disease. Animal models of AD have provided much information on the function of the basal forebrain system and have been used to investigate the potential effectiveness of various pharmacotherapies designed to reverse specific symptoms.

However, a model that recapitulates all the aspects of AD has not yet been produced, even if there are transgenic lines that offer robust and relatively faithful reproductions of a subset of its features. Nevertheless, one barrier to the efficiency of drug discovery efforts in the area of AD therapeutics is the time and labor intensive nature of animal studies using transgenic mice.

In light of these considerations, this review provides an extensive overview on several AD models, ranging from invertebrate to rodent animal models, and finally explores new perspectives, such as induced pluripotent stem cells and computational models.

## NON-RODENT MODELS OF ALZHEIMER’S DISEASE

### INVERTEBRATE MODELS

A majority of the genes linked to human disease, such as AD, belong to evolutionarily conserved pathways found in simpler organisms. The genes and pathways of these simple organisms can be genetically and pharmacologically manipulated to better understand the function of their orthologs *in vivo*, and to investigate how these genes are involved in the pathogenesis of different diseases. Often these manipulations can be performed much more rapidly in flies and worms than in mammals, and can generate high quality *in vivo* data that are translatable to mammalian systems. Other qualities also make these organisms particularly well-suited to the study of human disease. Invertebrate models are relatively inexpensive, easy to work with, have short lifespans, and often have very well-characterized and stereotypical development and behavior. This is particularly true for the two invertebrate model organisms: *Caenorhabditis elegans* and *Drosophila melanogaster*.

The modeling of age-related neurodegenerative diseases, such as AD, in invertebrate systems generally relies on two different methods: (1) genetic manipulation of the orthologs invertebrate genes that are associated with disease-causing pathways, and/or (2) expression of human versions of disease-related proteins in the model organism. Indeed, several pathways that play a vital role in normal development were first identified and investigated in *Drosophila* (Nusslein-Volhard and Wieschaus, 1980); also, genetic regulation of programmed cell death and RNA interference were first elucidated in *C. elegans* (Brenner, 1974; Ellis and Horvitz, 1986; Fire et al., 1998). Additionally, some of the major molecular pathways that regulate normal aging were found in both of these model organisms as well (Lin et al., 1998; Zou et al., 2000; Zhan et al., 2007; Smith et al., 2008), illustrating the power and productivity of studying age-related disease mechanisms in invertebrate systems.

While vertebrate models may provide a closer match to humans evolutionarily, invertebrate models can still provide useful information about disease progression and the function of genes involved in AD.

#### Analysis of invertebrate orthologs of AD genes

Most of the proteins associated with AD are evolutionarily conserved in *Drosophila* and *C. elegans*, making these organisms attractive model systems for understanding the conserved molecular functions of these genes. For instance, the ortholog of the human APP protein is *Apl-1* in *C. elegans* (Daigle and Li, 1993) and *Appl* in *Drosophila* (Rosen et al., 1989; Luo et al., 1990). In *C. elegans*, *Apl-1* is expressed in multiple tissues and is essential for viability (Hornsten et al., 2007). In *Drosophila*, *Appl* transcripts are mainly localized in the cortical region of the fly nervous system (Martin-Morris and White, 1990). Luo et al. (1992) showed that there is a functional conservation between fly Appl and human APP proteins, as the behavioral defects observed by a null mutant of fly Appl are rescued by expressing the human APP protein in these mutants. The functional ortholog of α-secretase in the fly is the *kuzbanian* gene (Rooke et al., 1996; Allinson et al., 2003). In *C. elegans*, there are two α-secretase orthologs, *sup-17* which is ortholog to mammalian *ADAM10* (Tax et al., 1997; Wen et al., 1997) and *adm-4* which is ortholog to mammalian *ADAM17/TACE* (Jarriault and Greenwald, 2005). To date, there is no evidence of β-secretase activity in *C. elegans* (Ewald and Li, 2010). Recently, however, a functional β-secretase ortholog was identified in *Drosophila* (Carmine-Simmen et al., 2009). Carmine-Simmen et al. (2009) found that the fly Appl protein can be cleaved by *Drosophila* BACE (dBACE) to generate a *Drosophila* Appl-specific Aβ peptide. This fly Aβ peptide also shows neurotoxic properties similar to human Aβ peptides (Carmine-Simmen et al., 2009), providing intriguing evidence to suggest conservation in the role of Aβ peptide-induced toxicity across species. Similarly, expression of either human or *Drosophila* α-cleaved ectodomains was shown to have neuroprotective effects in multiple fly neurodegeneration mutants (Wentzell et al., 2012), suggesting conserved functions of this domain across species as well. Each member of the mammalian γ-secretase complex (presenilin 1, presenilin 2, nicastrin, Aph-1, Pen-2; Kimberly et al., 2003) is also conserved in both flies and worms (Levitan and Greenwald, 1995, 1998; Hong and Koo, 1997; Li and Greenwald, 1997; Goutte et al., 2000; Wen et al., 2000; Chung and Struhl, 2001; Francis et al., 2002). Both flies and worms contain an ortholog of tau as well.

**FIGURE 2:**
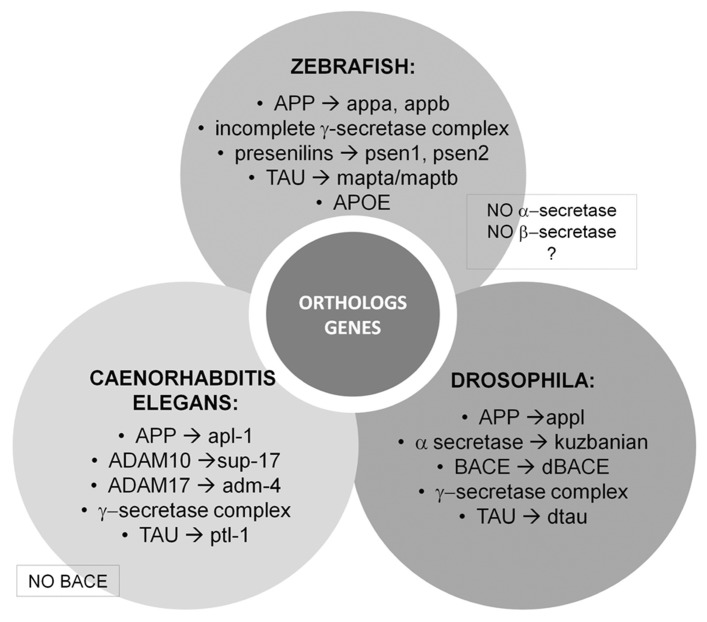
**Vertebrate and invertebrate orthologs genes.** Most of the proteins associated with AD are evolutionarily conserved in *Drosophila* and *Caenorhabditis elegans* making these organisms attractive model systems to better understand the conserved molecular functions of these genes linked to human disease. On the other hand, zebrafish (*Danio rerio*) is a promising model organism to study various central nervous system disorders, including AD.

In addition to the APP and the secretases genes, invertebrate models are useful tools to understand the function of genes that have been associated with increased risk for LOAD. Several genetic association studies have identified common variants of genes including *CLU* (clusterin), *BIN1* (bridging integrator 1), and *PICALM* (phosphatidylinositol binding clathrin assembly protein) as potential LOAD-related risk genes (Bertram et al., 2007). Invertebrate orthologs of these genes have been associated with cellular cytoskeletal dynamics, clathrin-mediated endocytosis, and postsynaptic exocytosis (Nonet et al., 1999; Mathew et al., 2003; Bao et al., 2005; Dickman et al., 2006). See **Figure 2** for a schematic representation.

#### Caenorhabditis elegans

*Caenorhabditis elegans* offers an efficient *in vivo* system to examine the toxic outcomes of overexpression of proteins and peptides that are prone to pathological misfolding (Teschendorf and Link, 2009). *C. elegans* can be further used as a cost-effective platform for discovering compounds that protect against the toxicity associated with these misfolded proteins. Simple animal models, like *C. elegans*, do not need to recapitulate all pathological aspects of the respective diseases. Indeed, the simplicity of this model may be advantageous; the potentially confounding behavioral and cognitive responses typical of the higher vertebrate are absent. Instead, rapid and clear toxic phenotypes may be preferable for screening strategies, facilitating identification of structure–activity relationships. The well-developed genetics and short life cycle of *C. elegans* allow it to be used in ways that are time and cost-prohibitive in vertebrate systems. As such *C. elegans* represents a complementary tool in drug discovery that may be employed before testing in vertebrate models, to expedite development of new therapeutics. To be useful for drug discovery this model must be predictive of efficacy in traditional vertebrate models.

*Caenorhabditis elegans* constitutes an excellent genetic system for studying molecular pathways involved in AD and tauopathies. Its capacity to express tau human proteins makes it a good animal model for these diseases. As mentioned above, the nematode has the gene *Apl-1*, which encodes two almost identical isoforms orthologs to the human APP, although it does not produce Aβ peptides because it lacks the β-secretase cleavage (Wentzell and Kretzschmar, 2010). Interestingly, earlier transgenic models of expressing human-Aβ in *C. elegans* do not accumulate the full length Aβ_1__-__42_ as expected, but predominantly a Aβ_3__-__42_ truncation product due to an aberrant cleavage of the N-terminal signal peptide incorporated in the *C. elegans* transgene. *In vitro* analysis demonstrates that Aβ_3__-__42_ self-aggregates like Aβ_1__-__42_, but more rapidly, and forms fibrillar structures. The Aβ_3__-__42_ peptide is also a more potent initiator of Aβ_1__-__40_ aggregation (McColl et al., 2009). To test the toxic effects of the Aβ_3-42_ a paralysis assay was done and *C. elegans* expressing Aβ_3-42_ showed an age-dependent paralysis phenotype. This finding makes *C. elegans* a good model to study the toxic effects and processing of Aβ_3__-__42_. However, Aβ_3-42_ does not significantly contribute to the Aβ found in human AD brain.

A *C. elegans* model expressing a more disease relevant form of Aβ is required in order to fully exploit this system for drug discovery. This model, expressing Aβ_1__-__42_, was generated by McColl et al. (2012). To achieve correct signal peptide cleavage from the Aβ_1__-__42_ they inserted two additional aa (-DA-) between the synthetic signal peptide and the Aβ peptide corrected the cleavage, resulting in expression of full length Aβ_1__-__42_. This model also provides a useful tool to explore the mechanism(s) of Aβ_1__-__42_ toxicity.

In light of the above, *C. elegans* is proving useful as an *in vivo* molecular genetic model for understanding basic mechanistic pathways of complex human neurological diseases as AD. The nematode can be used both as a monogenic model studying particular genes that are orthologs of human genes and as a versatile organism that allows transgenic expression of human genes. In addition, *C. elegans* makes available a well-characterized system in that neurodevelopment is known at the cellular level, making it possible to observe developmental features simultaneously with an analysis of molecular and behavioral phenotypes. Furthermore, since the nematode is only a millimeter long and its behavior can be monitored in liquid, it may be used as a system for whole-animal high-throughput screening, which might be helpful for finding new and specific clinical therapeutic drugs.

#### Droshophila

During the last decade, *Drosophila* has emerged as a powerful model to study human neurodegenerative diseases including AD. The short generation time (~10 days) and short lifespan (~60–80 days) make it particularly amenable to study such age-related disorders. Moreover, genome studies have revealed that ~70% of known disease-causing genes in humans are conserved in flies including several genes implicated in AD such as *APP* (*Appl*), *PSEN* (*Psn*), and *tau* (*tau*; Reiter et al., 2001). The development of *Drosophila* has also been extensively characterized and many tools are available to determine the effect of mutations on specific cell/tissue types, including neurons. Techniques to generate transgenic flies have also been established including several methods to express genes in a spatially and temporally restricted manner. Finally, synaptic activity can be measured using electrophysiological and imaging techniques from both the neuromuscular junction and more recently, the adult central nervous system (CNS). Genes identified as being potentially involved in AD progression can also be examined for their effects on various behaviors including locomotion and learning and memory. Various tools available in flies can be used to develop models that reproduce the key features of AD including plaques, tangles, memory defects, and death. As far as concern behavioral analysis, longevity measurement provides a statistically robust test of the neurological integrity of a fly. Although the cause of death is not clear, it is probably related to a combination of behavioral deficits that impair feeding and hazard avoidance (Crowther et al., 2006). In flies the most widely used behavioral assays that are employed are Pavlovian conditioning tests of memory and learning (Iijima et al., 2004, 2008) and locomotor assays (Iijima et al., 2004; Crowther et al., 2005, 2006; Rival et al., 2009). Subtle changes in locomotor behavior characterize the early stages of neuronal dysfunction, e.g., *Drosophila* models of AD show a significant decrease in climbing ability.

To date, the majority of models of AD in *Drosophila* have been based on the expression of APP or Aβ. Although the *Drosophila* genome encodes an APP ortholog, *Appl*, some critical elements to the pathological signature of AD are not as obviously conserved. Specifically, as reported in Section “Analysis of Invertebrate Orthologs of AD Genes,” *Drosophila* *Appl* does not contain a recognizable Aβ sequence and until recently no *BACE* ortholog has been identified. However, Carmine-Simmen et al. (2009) have recently identified a *Drosophila* BACE-like enzyme, dBACE. Moreover, overexpression of dBACE and APPL results in the production of a fragment containing the region in APPL that corresponds to the Aβ peptide, which accumulates in neurotoxic aggregates and induces age-dependent, AD-like behavioral deficits and neurodegeneration (Carmine-Simmen et al., 2009).

Many groups have also generated models of APP-mediated AD-like pathologies in flies by expressing transgenic constructs of wild-type and FAD human APP, in the presence or absence of other transgenes relevant to AD pathogenesis. For example, co-expression of human wild-type APP and BACE together with *Drosophila* Psn led to the production of Aβ peptides in flies and resulted in age-dependent neurodegeneration, accumulation of Aβ plaques and semi-lethality that could be suppressed by propagating the flies on BACE or γ-secretase inhibitors (Greeve et al., 2004). This phenotype could also be suppressed by loss of function mutations in *Psn*, while co-expression with FAD-associated *Psn* transgenes enhanced the neurodegeneration phenotype (Greeve et al., 2004), supporting an Aβ-related toxic gain of function for FAD-associated mutations. Stokin et al. (2008) co-expressed human FAD APP together with *Drosophila* FAD Psn in flies and observed increased levels of Aβ_1__-__42_ and enhanced amyloid deposition. Surprisingly, despite the presence of increased levels of Aβ_1__-__42_, they also observed suppression of the axonal transport defects and neuronal apoptosis in flies expressing FAD APP alone. This led them to conclude that the APP-induced axonal defects were not due to Aβ (Stokin et al., 2008).

Many groups have taken a more direct approach to study the role of Aβ_1__-__42_ in the onset and progression of AD by directly expressing the Aβ_1__-__42_ peptides in fly neurons. Importantly, unlike most mouse models of Aβ, transgenic flies expressing Aβ_1__-__42_ exhibit extensive cell loss, a difference that may be due to the fact that Aβ accumulates intracellularly within neurons in flies whereas they are extracellular in most mouse models. While the traditional clinical pathology of AD includes extracellular amyloid plaques, human AD brains show significant accumulation of intraneuronal Aβ_1__-__42_ (Takahashi et al., 2002). APP and Aβ_1__-__42_ expressing flies can thus recapitulate many important aspects of AD, including some phenotypes difficult to model in the mouse system, such as neuronal cell loss.

However, mutations in *APP* are a rare cause of FAD, with the majority of FAD-associated mutations occurring within the *PSEN* (Selkoe, 2001). Many groups have therefore sought to understand the genetic and biochemical role that presenilins plays in the onset and progression of AD. *Drosophila* models of presenilin-mediated AD phenotypes have provided an increased understanding of the role of presenilins in both development and disease. Mutations in conserved residues of *Drosophila* Psn have been used to model clinically heterogeneous human presenilins FAD mutations and to assess their activity. Since several attempts to model AD in flies have focused on studying the role of tau in the generation of NFTs, different groups have investigated the effect of expressing wild-type or mutant tau in various fly tissues.

Collectively these studies confirm critical roles for presenilins, APP, and Aβ_1__-__42_ in AD and demonstrate how *Drosophila* can be used as an effective tool to understand the onset and progression of AD-like phenotypes.

### ZEBRAFISH TO MODEL ALZHEIMER’S DISEASE PATHOLOGY

The zebrafish (*Danio rerio*) is rapidly emerging as a promising model organism to study various CNS disorders, including AD. Historically, rodent models have been used to study AD but additional animal models with complementary advantages must be used to analyze the basis of the neurodegeneration and, subsequently, to evaluate the effects of novel drugs, as a previous step to assays in rodents. Phenotypes of zebrafish at different development stages have been well-characterized, especially the development of the nervous system (Kimmel et al., 1995). Zebrafish offers a reasonable compromise between physiological complexity and throughput, has a fully characterized genome, and displays significant physiological homology to mammals, including humans (Esch et al., 1990). Moreover, it presents a clear advantage in comparison to other animal models: the larva–adult duality. The availability of both forms is beneficial and enables the investigation of a wider spectrum of neurodegenerative-related phenomena throughout ontogenesis.

The faster development and shorter lifespan of zebrafish, compared to mice, makes them and ideal choice to model developmental trajectories of neurodegeneration. Moreover, as rodent models are expensive to maintain and more difficult to modify genetically, lower organisms emerge as useful species to increase the knowledge of the mechanisms underlying neurodegenerative processes. The zebrafish is a vertebrate more closely related to humans than invertebrate models such as yeast, worms, or flies (Guo, 2004). Although invertebrates can provide important insights into neurodegeneration, the absence of a complex nervous system limits their application in modeling intricate aspects of CNS disorders. Comparison between various species is also important for uncovering mechanisms associated to brain pathogenesis. While the complementary use of zebrafish could be an important strategy for AD research, it represents an integral part of a more global cross-species modeling (from fish to rodents) for uncovering evolutionary conserved mechanisms of neurodegeneration. Furthermore, zebrafish has a short time of development to sexual maturity (3 months) and a high reproductive rate (hundreds of embryos per female per clutch and per week). Embryos are transparent and developed externally, facts that allow direct observation of embryogenesis and development of the CNS. Embryos are also easily amenable to methods for manipulating genes and protein activity such as injection of antisense oligonucleotides (Nasevicius and Ekker, 2000; Eisen and Smith, 2008), mRNAs or transgenes, and for screening of drug libraries being arrayed in microtiter plates (Zon and Peterson, 2005). Moreover, whereas the early zebrafish developmental stages are likely to be important to analyze CNS processes and abnormalities, adult fish with the full range of brain functions should have a significant advantage in the analysis of the complex brain functions characteristics of vertebrates (Panula et al., 2006), and in the studies of conductual phenotyping. Indeed, zebrafish exhibit many higher order behaviors including memory, conditioned responses, and social behaviors like schooling (Lieschke and Currie, 2007). All these properties strengthen the zebrafish as an ideal model for studying human diseases, including CNS disorders (Grunwald and Eisen, 2002). As reported before, one important advantage of the zebrafish is the genome organization and the genetic pathways controlling signal transduction and development, because they are highly conserved between zebrafish and vertebrates (Postlethwait et al., 2000). Indeed, the zebrafish genes share 50–80% homology with most human sequences and several genome databases similar to mouse have been generated. For these reasons, transgenic zebrafish are continuously increasing in number and being used to model human diseases.

Two orthologs of *APP*, *appa* and *appb* have been identified in zebrafish. Both genes have approximately 70% amino acid identity to human APP695, with 80% identity in the Aβ_1__-__42_ region and 95% identity within the transmembrane domain (Musa et al., 2001). The expression pattern of these two genes is similar to that observed for the mouse APP695 isoform (Sarasa et al., 2000). This indicates that APP gene function is conserved during vertebrate evolution. Regarding to APP zebrafish models, Lee and Cole (2007) used the zebrafish *appb* promoter to express green fluorescent protein (GFP) in transgenic animals. This model revealed that *appb* is mainly expressed in sub regions of brain, spinal cord, and the developing vasculature of the zebrafish embryos. Moreover, in adult transgenic zebrafish, *appb* was abundantly expressed in the brain (telencephalon, optic tectum, thalamus, and cerebellum; Lee and Cole, 2007). The demonstration that zebrafish *appb* gene regulatory elements can be used to drive GFP expression in both embryos and adults will be important for future studies that focus on expressing mutant human APP in zebrafish. In this study, the authors predicted that zebrafish expressing mutant human APP will generate Aβ plaques, although did not demonstrate their presence. In other study, Joshi and coworkers knocked down the zebrafish *appa* and *appb* function by morpholino (MO) injection resulting in a shortened body axis, a short, curly tail, mild synophthalmia, and defective convergent-extension movements during gastrulation (Joshi et al., 2009). Injecting *appa*-MO showed no or mild defects in developing embryos whereas *appb*-MO caused phenotypic changes similar to those observed when both MOs were injected together. These defects can be rescued by human APP695 mRNA, partially rescued by human sAPPα mRNA, but cannot be rescued by APP bearing the Swedish mutation (APP^swe^; (Joshi et al., 2009). These data suggest that APP processing plays an important role in APP function and that APP^swe^ is functionally deficient and the possibility that APP^swe^ may not only contribute to AD pathogenesis during aging but also may exert changes during embryonic development.

Despite conservation of the Aβ domain and of the secretases between zebrafish and humans, a zebrafish Aβ peptide has not yet been found and it is not known if the above posttranslational modifications that occur in human APP processing also occur in zebrafish.

Zebrafish embryos are a valuable system in which to study presenilins function. Orthologs of *PSEN1* and *PSEN2* have been identified in zebrafish (*psen1* and *psen2*). Sequence alignment of zebrafish and human presenilins protein sequences displays these primary structures as being highly conserved, though there are highly variable regions at the N-terminus and the C-terminal half of the cytoplasmic loop domains. Transcripts from zebrafish *psen1* are ubiquitously expressed from fertilization in the zebrafish. Zebrafish *psen2* mRNA is present from fertilization but protein expression has only been detected from the onset of gastrulation (6 h post fertilization). By modulating presenilins expression, it is possible to elucidate its function and regulation in zebrafish.

Regarding the tau zebrafish models recent developments strengthen the zebrafish as a model for the study of tauopathies (Bai and Burton, 2011) also because the zebrafish genome contains highly conserved orthologs of each of the kinases implicated in tau phosphorylation (Bai et al., 2007).

**FIGURE 3:**
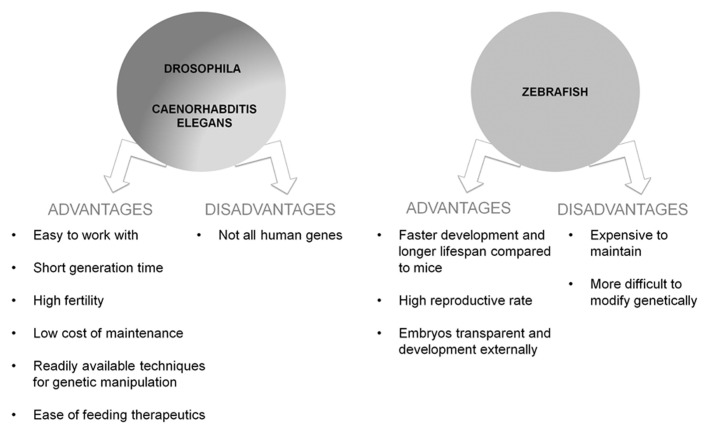
**Schematic representation of advantages and disadvantages of the use of the vertebrate and invertebrate models of AD**.

To date, the main genetic risk factor associated with the sporadic form of AD is the allele 4 of the APOE gene (APOE-ε4; Corder et al., 1993; Saunders et al., 1993). A zebrafish gene ortholog to mammalian *APOE* has been previously identified (Durliat et al., 2000) but APOE zebrafish transgenic models have not yet been developed. For a summary of advantages and disadvantages of vertebrate and invertebrate models see **Figure 3**.

## RODENT MODELS OF ALZHEIMER’S DISEASE

The mouse was the first organism in which transgenic technology and gene targeting became widely available. Mice offer several advantages in scientific research: they are small and easy to rear, their progeny are abundant and gestation very short and last, but not least, their genome was whole mapped. A valid animal model for AD should exhibit progressive disease-like neuropathology and cognitive deficits; reliable models should manifest little cognitive deficits until a certain advanced age and then should ideally display progressive impairment of specific memory system while other memory systems are saved.

A mouse model that recapitulates all the aspects of AD has not yet been produced, even if each model allows for in-depth analysis of one or two components of the disease, which is not readily possible or ethical with human patients or sample.

### TRANSGENIC ALZHEIMER’S DISEASE MODELS

In light of the importance of creating a reliable AD model, several transgenic mice lines were generated. These models should be able to discern pathogenic effects of familiar mutations from those of transgene overexpression (Janus and Westaway, 2001).

Since 1993, numerous knockout animals have been developed to explore a particular aspect of AD: suppression of genes that encode proteins involved in the pathogenesis has helped to anticipate the effects of presumptive new molecules designed to regulate these protein. Knockout mice have been made not only of the APP, secretases, i.e., BACE, PSEN1 and 2, ADAM10 (Shen et al., 1997; Herreman et al., 1999; Luo et al., 2001; Hartmann et al., 2002; Lee et al., 2003), but also of both APP and tau protein, proving invaluable in understanding disease progression and in identifying physiological roles for the APP protein (Zheng et al., 1996; Takei et al., 2000)*.* Presenilin knockout mice developed striking neurodegeneration of cerebral cortex, impairment of memory and synaptic function, which call for caution in the therapeutic use of γ-secretase inhibitors (Shen et al., 1997; Saura et al., 2004). BACE is the exclusive β-secretase enzyme, and its activity is also crucial for the production of Aβ. Although its physiological functions were unclear, this enzyme has been object of inhibitor program; β-secretase knockout mice are perfectly viable with no obvious defects and no longer produced any Aβ (Luo et al., 2001; Roberds et al., 2001). In 2006, Hu and Willem reported that BACE knockout mice showed significantly reduced level of myelination and myelin thickness (Hu et al., 2006; Willem et al., 2006), suggesting an important role of this enzyme in the development of the nervous system. Furthermore, BACE has a large active site, which makes difficult to find compounds that cross blood–brain barrier and are large enough to inhibit the active site without being hydrolyzed by endopeptidase (Huang et al., 2009).

Current transgenic models of AD rely on information gathered from inherited forms of the disease, far less common than sporadic ones but indistinguishable from a clinical histopathological point of view. A first attempt to generate transgenic model counted only on overexpression of entire sequence of human APP770 gene (Buxbaum et al., 1993; Lamb et al., 1993), human APP751 (Quon et al., 1991; Moran et al., 1995), human APP695 (Yamaguchi et al., 1991), and Aβ (Wirak et al., 1991) in the brain. Despite APP transgene was successfully expressed in the brain, these models did not provide evidence of plaque deposition in most cases, and displayed very mild neuropathological changes. The lack of plaques in these transgenic mice was generally attributed to insufficient levels of the transgene, to the absences of a disease-causing mutation in the transgene, to the genetic background of mice or to other unknown factors inherent to the mouse brain, compared to human. The finding that besides its overexpression APP must be also mutated for the production of high levels of Aβ led later to generation of new animal models; these mice developed age-dependent AD-like pathology including amyloid deposit in brain parenchyma (Hsiao et al., 1996; Moechars et al., 1999). Of the more than 20 autosomal dominant APP mutations linked to AD that appear to enhance the aggregation of Aβ, only few mutations have been used to generate transgenic mice: V717I “London” mutation (Goate et al., 1991), V717F “Indiana” mutation (Murrell et al., 1991), K670D/M671L “Swedish” mutation (Mullan et al., 1992), and E693G “Artic” mutation (Nilsberth et al., 2001).

However, transgenic mice do not develop extensive neuronal loss, like human AD patients. This can be ascribed to the amount of time needed: in human AD progresses over decades while transgenic mice are kept only for 2 years. Another problem may be related to background strains: (i) the most used mice strain is C57BL/6 which may be more resistant to excitotoxicity, (ii) the heterogeneity of humans is more complex compared with inbred mouse strains, (iii) fundamental differences between mice and humans exist (LaFerla and Green, 2012).

Here we report a description of the main characteristics of the most used transgenic mice to reproduce AD.

#### PDAPP mice

Report of the first transgenic mouse able to develop a robust AD-related phenotype was published in 1995 (Games et al., 1995). This line [known as PDAPP (PDGF-hAPP), **Figure 4A**] overexpresses human APP with “Indiana” mutation under the control of platelet-derived growth factor beta (PDGF-β) promoter on a mixed C57BL/6, DBA, and Swiss-Webster strain background, which leads to a 10-fold elevation of APP sufficient to generate Aβ for extracellular plaques. The PDGF-β promoter targets expression preferentially to neurons in the cortex, hippocampus, hypothalamus, and cerebellum of the transgenic animals. The PDAPP model was the first transgenic mouse to overexpress the human APP that successfully recapitulated several neuropathological features characteristic of AD: Aβ deposition in both diffuse and neuritic plaques, cerebral amyloid angiopathy (CAA), astrocyosis, microgliosis, hippocampal atrophy, synaptic alterations, and behavioral deficits (Dodart et al., 1999). Many of the histological, biochemical, and structural alterations of PDAPP mouse closely resemble the changes found in the brain of AD patients, especially the temporal and spatial-specific deposition of Aβ in the brain. The animals, however, did not develop tauopathy or significant neuronal cell death (Irizarry et al., 1997b). At 3 month of age the transgenic mice displayed significant hippocampal atrophy, while at 4–5 months of age showed an enhanced paired-pulse facilitation (PPF), distorted responses to high frequency stimulation bursts, and long-term potentiation (LTP) decays more rapidly (Larson et al., 1999). In heterozygotic mice, between 4 and 6 months of age, no obvious pathology was detected; however, at ~6–9 months of age, transgenic animals began to exhibit deposits of human Aβ in the hippocampus, corpus callosum, and cerebral cortex, but not in other brain regions. These increased with age, and by 8 months, many deposits were seen. At age >9 months, the density of the plaques increased until the Aβ-staining pattern resembled that of AD. By 18 months, they produced neuritic alterations and gliosis without widespread neuronal death (Irizarry et al., 1997b; Johnson-Wood et al., 1997). The PDAPP mouse has proven to be an attractive model to study the disease process underlying aspects of AD that are related to Aβ aggregation and its consequences. Although this model developed amyloid deposits, it failed to meet all criteria of the neuropathology of human AD in the absence of cortical or hippocampal neuronal loss or NFTs in aged transgenic animals (Irizarry et al., 1997a). The PDAPP mouse has been extensively used to study the effect of genetic factors that modify AD, as well as Aβ-binding proteins and their effect on Aβ deposition (Brendza et al., 2002).

**FIGURE 1:**
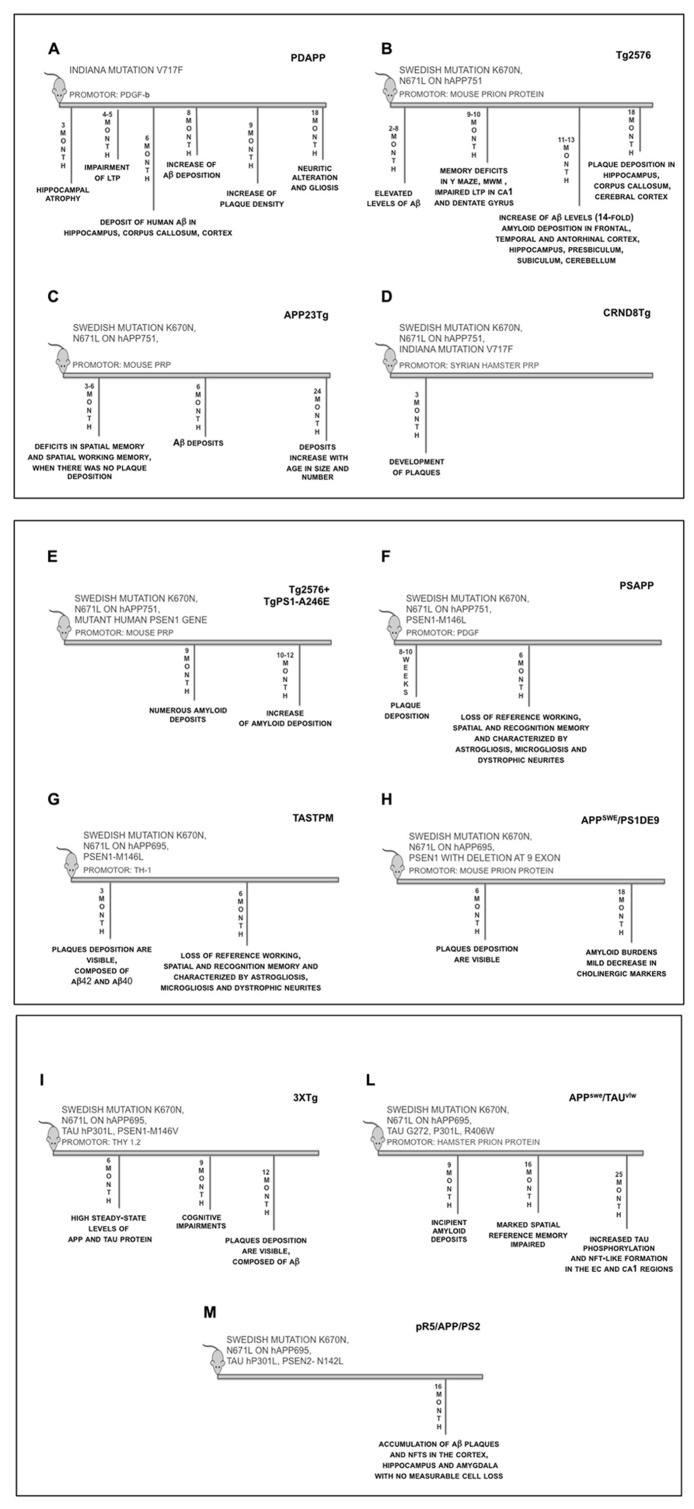
**Time-line of the main features of transgenic mice.** **(A)** PDAPP mice overexpress human APP transgene containing the Indiana mutation (V717F) under the control of the PDGF-β promoter; **(B)** Tg2576 mice overexpress human APP695 transgene containing the Swedish mutation (APPK670/671L) under the control of the hamster prion protein promoter; **(C)** APP23Tg mice overexpress human APP751 transgene containing the Swedish mutation (APPK670/671L) under the control of Thy-1.2 promoter; **(D)** CRND8Tg mice overexpresses human APP695 transgene containing the Swedish (APPK670/671L) and Indiana mutation (V717F), under the control of Syrian hamster PrP promoter; **(E)** Tg2576 mice were crossed with Tg A246E mice (expressing a mutant human PSEN1 gene), under the control of the mouse prion protein promoter; **(F)** Tg2576 was crossed with mice expressing human PSEN1-M146L mutant, under PDGF promoter; **(G)** TASTPM mice express human APP with Swedish mutation (APPK670/671L) and PS1-M146V mutation, under Thy-1 promoter; **(H)** APP^swe^/PSEN1dE9 mice express human APP with Swedish mutation (APPK670/671L) and PSEN1 with exon 9 deletion, under mouse PrP promoter; **(I)** 3xTg mice: in this model human APP695 cDNA harbouring the Swedish mutation (KM670/671NL) was subcloned into exon 3 of the Thy1.2 expression cassette. Human four-repeat tau without amino terminal inserts (4R0N) harbouring the hP301L mutation was also subcloned into Thy1.2 expression cassette. The two were comicroinjected into the pronuclei of single-cell embryos harvested from homozygous PSEN1-M146V knockin mice; **(L)** APP^swe^/TAU ^vlw^ mice areobtained by crossing Tg2576 mice and TAU ^vlw^ mouse lines expressing human 4-repeat tau containing a triple mutation (G272V, P301L and R406W) and both gene are under control of hamster PrP promoter; **(M)** pR5/APP/PSEN2 triple transgenic**: **APP^swe^PSEN2 ^N141I^ double transgenic mice were crossed with P301L tau transgenic pR5 mice

#### Tg2576 mice

In 1996, a second line (Tg2576, **Figure 4B**), created by Hsiao et al. (1996), also made sufficient Aβto form deposits, and, in addition, this mouse showed age-related cognitive impairment. Human APP695 containing the double mutation Lys670->Asn, Met671->Leu (Swedish mutation), was inserted into B6;SJL F2 mice using a hamster prion protein cosmid vector. The resultant transgenic mice were bred to C57BL/6 mice. The colony was maintained by mating hemizygotes to B6SJL F1 mice. Transgenic mice 11–13 months of age show a 14-fold increase in Aβ_1__-__42/43_ over those at 2–8 months of age. The elevated Aβ levels are associated with the development of amyloid deposits in frontal, temporal, and entorhinal cortex (EC), hippocampus, presubiculum, subiculum, and cerebellum (Hsiao et al., 1996). Nine to ten month old Tg2576 mice demonstrate memory deficits in Y mazes and Morris water maze (MWM) behavioral tests. These behavior alterations correlate with the development of amyloid plaques and with impaired LTP in both the CA1 and dentate gyrus regions of the hippocampus (Chapman et al., 1999). These transgenic mice also show a significant microglial response to the amyloid plaques with an increase in both number and area (Frautschy et al., 1998). Although several groups have described cognitive decline with the appearance of senile plaques (Chen et al., 2000; Janus et al., 2000; Gordon et al., 2001), deep cognitive investigation into Tg2576 mice over a broader age range (4–22 months), failed to show any overall correlation between memory function and insoluble Aβ levels, although correlation was observed within individual age groups (Westerman et al., 2002). Characterization of the timing and nature of preplaque dysfunction is important for understanding the progression of this disease and to identify pathways and molecular targets for therapeutic intervention. Examination of the progression of dysfunction at the morphological, functional, and behavioral levels in the Tg2576 mouse model of AD, shows that decreased dendritic spine density, impaired LTP, and behavioral deficits occurred months before plaque deposition, which was first detectable at 18 months of age (Jacobsen et al., 2006). As for PDAPP model, Tg2576 mice do not exhibit neuronal loss despite having extensive amyloid deposition (Irizarry et al., 1997b).

#### APP23Tg mice

Another mouse model similar to Tg2676 is APP23Tg mouse (**Figure 4C**). This mice line was constructed on a C57BL/6//DBA/2 genetic background (Andra et al., 1996); human APP751 with Swedish mutation was insert under control of Thy-1.2 promoter. Mice have a sevenfolds overexpression of APP and first show Aβ deposits at 6 months of age (Sturchler-Pierrat et al., 1997). Deposits increase with age in size and number and eventually occupy a substantial area of the neocortex and hippocampus in 24 months old mice. Associated with the amyloid, there are inflammatory reactions, neuritic and synaptic degeneration as well as tau hyperphosphorylation. The plaque cores are substantially more soluble than those in the AD brain. The mice show more congophilic plaque, high levels of Aβ in cerebrospinal fluid, and regional localization of CAA (Winkler et al., 2002). Cerebral amyloidosis is not sufficient to account for global synapse loss in AD (Boncristiano et al., 2005). Behavioral characterization was done at 3, 18, and 25 months of age (Kelly et al., 2003); in passive avoidance APP23Tg mice were unimpaired up to 25 months of age and, in spatial memory performance in the MWM test, the mice show age-dependent learning deficits and poor memory. Deficits in spatial memory and spatial working memory were found already at 3 and 6 months of age, when there was no plaque deposition (Van Dam et al., 2003). As in AD, no deposits are found in the cerebellum, but a considerable amount of vascular amyloid is detected. This is particularly noteworthy, because APP expression is restricted to neurons (Bornemann and Staufenbiel, 2000). All deposits show immunoreactivity with a number of different Aβ antibodies, which is comparable to the staining of brain sections from AD patients. The Aβ deposits also react with end-specific antibodies recognizing the amino terminus of Aβ or its carboxy termini at amino acids 40, 42, and weakly at 43 (Paganetti et al., 1996). Biochemical analyses of brain cortex lysates showed that the Aβ_1__-__40_ form is a little more prominent than the Aβ_1__-__42_ form. However, the amount of both Aβ_1__-__40_ and Aβ_1__-__42_ exponentially increases during aging in parallel to the increase in plaque volume, whereas the APP level remains constant (Bornemann and Staufenbiel, 2000).

#### CRND8Tg – TgJ9 – TgJ20 mice

In 2001, a transgenic line of the earliest-onset AD, that develops plaque as early as 3 months, was generated. These mice, named CRND8Tg, carry both the Swedish and Indiana mutation, and the human APP695 is under the control of Syrian hamster PrP promoter (Chishti et al., 2001). Expression of mutant APP is about fivefold than endogenous murine APP, and due to a rapid increase in the Aβ_1__-__42_/Aβ_1__-__40_ ratio, CRND8Tg develop a very aggressive pathology with a high mortality. To detect cognitive deficits different behavioral tests were carried out; in MWM (Chishti et al., 2001), fear conditioning (Greco et al., 2010), auditory startle, and prepulse inhibition (McCool et al., 2003) mice show impairments in reference, recognition, working, and spatial memory (**Figure 4D**).

Two others transgenic mice similar to CRND8Tg were generated: TgJ9 and TgJ20. These lines express both the Swedish and Indiana mutation on the human APP770 isoform under control of PDGF promoter. Unlike CRND8Tg, the pathology is less aggressive, in fact TgJ9 and TgJ20 show plaques deposition occurring at 8–10 months of age in the dentate gyrus and in the neocortex, furthermore expression of Aβ is low in TgJ9 mice rather than TgJ20 (Mucke et al., 2000; Chin et al., 2005). Although all transgenic mice described above do not recapitulate a complete picture of the neuropathology of AD due to absence of NFTs, neurodegeneration and cerebral atrophy, each model shows anatomical and morphological pattern of plaques formation similar to that seen in human AD.

#### Double transgenic mice

As for APP, mutations in presenilin are involved in FAD. To determine the effects of PSEN1 on APP processing, both wild-type and mutant presenilin 1 transgenic mice have been created (Duff et al., 1996). Transgenic mice expressing either a wild-type or mutated presenilin gene fail to develop significant AD-like pathology, despite having intra- but not extracellular Aβ deposition (Borchelt et al., 1996; Duff et al., 1996; Chui et al., 1999). These properties have prompted a number of studies into the characteristics of mice expressing the human versions of presenilins and of APP. To accelerate plaque deposition in transgenic line of AD, multiple PSEN1 and APP mutations were inserted in mice. Tg2576 mice were crossed with mice expressing a mutant human *PSEN1* gene (Tg A246E); the mouse prion protein promoter directs expression of both transgenes. Elevated levels of the Aβ_1__-__42/__43_ peptides are detected in brain homogenates. By 9 months of age, histological examination of brain tissue reveals numerous amyloid deposits resembling those observed in the brains of AD patients. The number of amyloid deposits increases dramatically between the ages of 10 and 12 months (**Figure 4E**). These mice provide a useful model for studying the underlying mechanism of amyloid deposition, a process implicated in AD (Borchelt et al., 1998).

When Tg2576 was crossed with mice expressing human PSEN1-M146L mutant under PDGF promoter, generating the PSAPP transgenic line (**Figure 4F**; Holcomb et al., 1998), plaque deposition occurred at 8–10 weeks compared to 8–12 months for single APP-mutated mice (McGowan et al., 1999; Kurt et al., 2001). Furthermore, double transgenic mice revealed a selective 41% increase in Aβ_1__-__42/43_ in homogenate of their brain. This model shows cognitive impairments due to loss of reference, working, spatial and recognition memory, and in addiction is characterized by astrogliosis, microgliosis and dystrophic neuritics (Holcomb et al., 1998). Another mouse model with the same histopathological characteristics of PSAPP is the TASTPM mouse (**Figure 4G**) that expresses human APP with Swedish mutation and PSEN1 with M146V mutation under Thy-1 promoter (Howlett et al., 2004, 2008). Plaque depositions are visible at 3 months of age and are composed of Aβ_1__-__42_ and Aβ_1__-__4__0_ with compact structure in cortical and hippocampal areas and localized neuronal cell loss (McGowan et al., 1999; Howlett et al., 2004). Single mutation insertion is not the only strategy used to manipulate *PSEN1* gene; *PSEN1* with deletion at exon 9 was co-expressed with APP Swedish mutant under mouse PrP promoter to generate APP^swe^/PSEN1dE9 (Lee et al., 1997). In this transgenic mice plaques appear less dense as in human pathology and have been found cognitive deficits in spatial and recognition memory (Howlett et al., 2004; Garcia-Alloza et al., 2006; Dinamarca et al., 2008). Spatial reference memory was assessed in a standard MWM task followed by assessment of episodic-like memory in Repeated Reversal and Radial Water maze tasks. At 6 months of age, APP^swe^/PSEN1dE9 double-transgenic mice showed visible plaque deposition (**Figure 4H**); however, all genotypes, including double-transgenic mice, were indistinguishable from non-transgenic animals (Savonenko et al., 2005). In the 18-month-old cohorts, amyloid burdens were much higher in APP^swe^/PSEN1dE9 mice with statistically significant but mild decreases in cholinergic markers (cortex and hippocampus) and somatostatin levels (cortex; Savonenko et al., 2005). Some forms of Aβ associated with amyloid deposition can disrupt cognitive circuits when the cholinergic and somatostatinergic systems remain relatively intact.

#### Tau pathology and triple transgenic mice

One of the limit of the transgenic mice described above, is the lack of NFTs even though the tau protein is present in hyperphosphorylated form. To exceed this, mice lines with mutations in tau protein or triple transgenic mice were created. In (Oddo et al., 2003b) generated a triple transgenic line (3xTg, **Figure 4I**): human APP695 cDNA harboring the Swedish double mutation (KM670/671NL) was subcloned into exon 3 of the Thy1.2 expression cassette. Human four-repeat tau without amino terminal inserts (4R0N) harboring the hP301L mutation was also subcloned into Thy1.2 expression cassette. The two were co-microinjected into the pronuclei of single-cell embryos harvested from homozygous PSEN1-M146V knockin mice (Oddo et al., 2003b). 3xTg mice develop an age-related and progressive phenotype including plaques and tangles. Synaptic dysfunction, including LTP deficits, manifests in an age-related manner, but before plaque and tangle pathology. Deficits in long-term synaptic plasticity correlate with the accumulation of intraneuronal Aβ (Oddo et al., 2006; Pietropaolo et al., 2008). Hippocampus and cerebral cortex contained highest steady-state levels of APP and Tau proteins and extracellular Aβ deposits first appear in 6-month-old mice in the frontal cortex, and were readily evident by 12 months. There is a progressive increase in Aβ formation as a function of age in the 3xTg brains and a particularly pronounced effect on Aβ_1__-__42_ levels (Oddo et al., 2003a). The mice develop cognitive impairments by 4 months of age. The first impairments manifest as a retention/retrieval deficit and not as a learning deficit, and occur prior to the occurrence of any plaque or tangle pathology. The mice show deficits on both spatial and contextual based paradigms and this early cognitive deficits can be reversed by immunotherapy (Oddo et al., 2004; Billings et al., 2005). Others transgenic lines with tau pathology were made; Tg2576 mice were crossed with tau^vlw^ mouse lines expressing human 4-repeat tau containing a triple mutation (G272V, P301L, and R406W) and both gene are under control of hamster PrP promoter (**Figure 4L**). The double transgenic mouse APP^swe^/tau^vlw^ (Perez et al., 2005) showed enhanced amyloid deposition accompanied by neurofibrillary degeneration and overt neuronal loss in selectively vulnerable brain limbic areas. Not only resulted in increased tau phosphorylation but also triggered NFT-like formation were observed in the EC and CA1 regions at 25 months of age (Ribe et al., 2005). Fivefold higher amyloid burdens appeared in APP^swe^/tau^vlw^ female mice in comparison to male mice. Co-expression of human mutant APP and tau in the APP^swe^/tau^vlw^ mouse model enhanced both AD and tau pathology and resulted in significant neuronal loss in selective vulnerable brain areas. Learning and memory deficits, as measured by the MWM test, were quite modest at 9 months of age despite incipient amyloid deposits in APP^swe^/tau^vlw^ and APP^swe^ brains, and significant neuronal loss in the EC in the former. By 16 months, however, both APP^swe^ and APP^swe^/tau^vlw^ mice showed marked spatial reference memory impairment compared to tau^vlw^ and non-transgenic littermate controls (Perez et al., 2005; Ribe et al., 2005).

Recently a new triple transgene line was generated: APP^swe^PSEN2-N141I double transgenic mice were crossed with P301L tau transgenic pR5 mice giving raise to pR5/APP/PSEN2 triple transgenic mice (Rhein et al., 2009; **Figure 4M**). This new model shows age-dependent accumulation of Aβ plaques and NTFs in the cortex, hippocampus and amygdala with no measurable cell loss at 16 months (Rhein et al., 2009; Grueninger et al., 2010).

### TgF344-AD RAT MODEL

Recently Cohen et al. (2013) have generated a transgenic rat model (TgF344-AD) expressing human APP with Swedish mutation and PSEN1 deleted in exon 9 (PSEN1ΔE9). The human genes, driven by mouse prion promoter, were co-injected in rat pronuclei on a Fischer344 background. TgF344-AD rats expressed 2.6-fold higher human holo- and secreted APP^SWE^ proteins than endogenous rat APP and 6.2-fold increased human PSEN1ΔE9 protein versus endogenous rat protein. The model manifests progressive age-dependent Aβ deposition not only in the hippocampus but also in the striatum and cerebellum. The authors have also assessed cognitive performance in the Barnes maze, a widely accepted test for hippocampus-dependent spatial reference learning and memory in rat (Barnes, 1979; Barnes et al., 1994); they found that 15-month-old Tg animals made significantly more errors during the learning phase versus 6-month-old Tg or wild-type animals. Moreover, Tg rats showed age-dependent abnormalities in open field activity and spatial learning memory. Unlike the transgenic mice that show tau pathology only when they harbor the mutated tau gene (Oddo et al., 2003b; Rhein et al., 2009), the TgF344-AD line manifests tauopathy relying solely on endogenous rat tau protein. Finally, Tg rats show consistent and extensive neuronal loss in cortical and hippocampal regions and an age-dependent trend toward decreased hemispheric brain weight (Cohen et al., 2013). In conclusion this model recapitulates all aspects of AD pathology and represents a starting point for a next-generation animal model to enable basic and translational AD research.

### NON-TRANSGENIC MICE MODELS OF ALZHEIMER’S DISEASE

None of the available models has helped to reach a comprehensive explanation of the origin of the disease, especially regarding sporadic LOAD and the complexity of its etiology. For this reason, new hypothesis on the origin of the disease and their corresponding animal models are blooming. For instance, loss of function of presenilin has been claimed to explain both FAD and sporadic LOAD (Shen and Kelleher, 2007) and other processes, such the relation between ApoE4 and cholesterol metabolism or the role of cholesterol, have been proposed as causative rather than progression factors in the pathological cascade. Published data show that senescence-accelerated mouse (SAMP8), a model of aging, displays many features that are known to occur early in the pathogenesis of the disease such as increased oxidative stress, Aβ alterations and tau phosphorylation (Tomobe and Nomura, 2009). Therefore, SAMP8 mice may be an excellent model for studying the earliest neurodegenerative changes associated with sporadic LOAD and provide a more encompassing picture of human disease, a syndrome triggered by a combination of age-related events and not-completely known risk factors.

Starting from the consideration that cerebral glucose metabolism abnormalities have been found in AD (Hoyer, 1992), models of AD-type neurodegeneration in adults rats were generated treating animals with intracerebroventricular injection of Streptozotocin (icv-STZ), a glucosamine–nitrosourea compound which generates a cytotoxic product when metabolized and causes oxidative stress, DNA damage, and diabetes mellitus, even if the mechanism of cytotoxicity is unknown (Plaschke and Hoyer, 1993; Duelli et al., 1994; Lester-Coll et al., 2006; Plaschke et al., 2010). The treatment causes chronic reduction in glucose and glycogen metabolism in the cerebral cortex and hippocampus (Plaschke and Hoyer, 1993) and these effects are associated with significant inhibition of insulin receptor function and progressive deficits in learning, memory, cognitive behavior, cerebral energy balance (Lannert and Hoyer, 1998; Hoyer and Lannert, 1999; Hoyer et al., 2000). Moreover, the treatment of rat pups with icv-STZ injection showed an alteration of insulin and insulin-like growth factor signaling mechanisms in the brain, an increase of phopsho-tau and extracellular plaque-like deposit of Aβ (Lester-Coll et al., 2006). Recently, the role of insulin resistant brain state (IRBS) was evaluated in genetic mice models of AD (Plaschke et al., 2010). Six months after icv-STZ treatment, nine-month-old Tg2576 mice were investigated: they showed an increase of mortality, reduction of spatial cognition, increase of cerebral aggregated Aβ_40__-__42_ fragments, total tau protein and congophilic amyloid deposits compared with mice treated with placebo (Plaschke et al., 2010). The authors showed for the first time that AD-like functional and structural changes in Tg2576 mice are aggravated by IRBS caused by icv-STZ injection, and these data support the concept that AD-type neurodegeneration represents an intrinsic neuroendocrine disease (Lester-Coll et al., 2006; Plaschke et al., 2010).

Another way to mimic AD is to perform icv injection of soluble Aβ_1__-__42_ (icv-Aβ) since it induces synaptotoxicity and memory dysfunction (Hardy and Selkoe, 2002), and several groups studied the effects on different pathways. Canas et al. (2009) showed that icv-Aβ caused a delayed loss of memory performance that was selectively associated with loss of synaptic markers without neuronal damage nor astrogliosis/microgliosis. Moreover, they demonstrated that blockade (pharmacologic or genetic) of adenosine A2A receptor (A2AR) can prevent this effect through a mechanism involving the control of p38 mitogen-activated protein kinase pathway. Lee et al. (2012) demonstrated that icv-Aβ in mice induced neuronal cell death in the hippocampal CA1 region, microglia and astrocyte activation, nitrotyrosine formation, inducible nitric oxide synthase expression, and memory impairment.

Even if these models do not recapitulate all the aspects of AD, they might be useful to study the different pathways affected in AD pathology, leading to the identification of potential therapeutic strategies.

**FIGURE 5:**
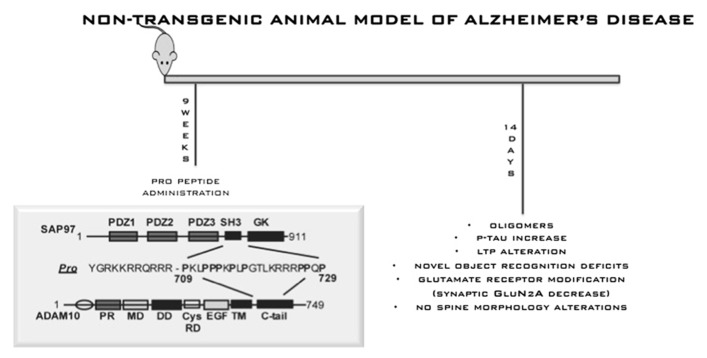
**Non-transgenic animal model for AD.** This model mimics early stages of sporadic disease, which represents the vast majority of cases. The model was obtained by interfering with the complex ADAM10/SAP97 for 2 weeks by means of a cell-permeable peptide strategy. This is sufficient to shift the metabolism of APP toward amyloidogenesis and allows the reproduction of initial phases of sporadic AD.

In this scenario, it is fundamental to generate new hypothesis. Recently, Kim et al. (2009) reported two potentially pathogenic mutations with incomplete penetrance for LOAD in the gene for the α-secretase ADAM10. It was already known that ADAM10 interacts directly with synapse-associated protein 97 (SAP97), a cargo protein involved in trafficking of glutamate receptors to the postsynaptic density. This interaction is required for ADAM10 localization at postsynaptic membranes and for its enzymatic activity (Marcello et al., 2007). As a consequence, interfering with ADAM10/SAP97 complex in rodents can reduce ADAM10 localization and activity on APP at synaptic membrane. This shifts APP metabolism toward amyloidogenesis. Given the identification of ADAM10 as a candidate AD susceptibility gene, interfering with its trafficking and with its activity could represent a new potential pathogenic mechanism that should be investigated in minute details in new natural mouse models. To this, we described an innovative, non-transgenic animal model of AD (Epis et al., 2010). This model mimics early stages of sporadic disease, which represents the vast majority of cases. The model was obtained by interfering with the complex ADAM10/SAP97 for 2 weeks by means of a cell-permeable peptide strategy. This is sufficient to shift the metabolism of APP toward amyloidogenesis and allows the reproduction of initial phases of sporadic AD (**Figure 5**). After 2 weeks of treatment, we detected progressive AD-like neuropathology, with an increase of Aβ aggregate production and of tau hyperphosphorylation, and a selective alteration of *N*-methyl-D-aspartic acid receptor subunit composition in the postsynaptic compartment of mouse brain. Behavioral and electrophysiological deficits were also induced by peptide treatment. Despite the important achievements obtained through transgenic mice, identifying the earliest possible mechanisms that induce evidence of neuropathology, before signs of functional deficits emerge, is now seen as a need. In this way, the most vulnerable neurons could be identified, new disease mechanisms could be investigated and a means of refining novel treatments would be provided. And eventual discrepancies and similarities between future models should not disqualify or diminish the importance of any mouse model but rather should stimulate more refined comparison.

## FUTURE PERSPECTIVES

### MODELING ALZHEIMER’S DISEASE IN CELLULAR SYSTEMS

Generation of human induced pluripotent stem cells (iPSCs) provides a new method for elucidating the molecular basis of human disease (Takahashi et al., 2007; Yu et al., 2007). An increasing number of studies have employed disease-specific human iPSCs in neurological diseases, and some of them have demonstrated disease-specific phenotypes to model the neurological phenotype (Dimos et al., 2008; Park et al., 2008; Ebert et al., 2009; Lee et al., 2009; Soldner et al., 2009; Chamberlain et al., 2010; Ku et al., 2010; Marchetto et al., 2010; Nguyen et al., 2011). The idea of studying AD with iPSCs initially seems counterintuitive. Taking a cell type that mimics the earliest stages of human development and expecting these cells to inform one about a disease that typically afflicts people over the age of 65 does initially seem at odds with our basic understanding of AD. APP, presenilin, and APOE transgenic and knockout mouse models have clearly taught us a great deal about the function of these genes and their role in AD pathogenesis. However, it is reasonable to conclude that genetic differences between humans and mice have also contributed to our difficulties in accurately modeling AD and generating clinically predictive data. Human stem cell-based models may therefore provide an alternative approach to clarify both the normal and pathogenic roles of AD-associated genes. Patient-derived iPSCs have been used to model a growing list of human genetic disorders (Grskovic et al., 2011).

Most recently, two groups have reported the establishment and investigation of AD iPSCs (Yagi et al., 2011; Israel et al., 2012). In one study, Yagi et al. (2011) generated iPSC from fibroblasts of FAD with the PSEN1 mutation A246E and the PSEN2 mutation N141I, and reported the differentiation of these cells into neurons. They demonstrated that patient-derived differentiated neurons show increased Aβ_1__-__42_ secretion, recapitulating the pathological mechanism of FAD with PSEN1 and PSEN2 mutations. Their findings demonstrated that the FAD–iPSC-derived neuron is a valid model for studying AD, and provides important clues for the identification and validation of candidate drugs. In a second study, Israel et al. (2012) generated iPSCs from not only FAD patients but also two cases of sporadic AD and two unaffected controls. Interestingly, neurons derived from one of the sporadic AD cases mimicked some of the findings from FAD cases; showing increased Aβ_1__-__40_ generation and tau phosphorylation, activation of glycogen synthase kinase-3b, and accumulation of enlarged early endosomes. Importantly, these disease-associated phenotypes were not detected in fibroblast cultures from this case, demonstrating the importance of studying these phenotypes in iPSC-derived neurons. Taken together, these studies represent critical first steps in assessing the potential of AD iPSCs to model AD. As Israel et al. (2012) point out, generation of many additional sporadic AD iPSC lines will of course be needed to fully establish the ability of this approach to guide drug development and enhance our understanding of AD. The generation of directly reprogrammed induced neural stem cells from AD patients may also help to accelerate such research.

Another way to study neurological diseases, such as AD, is the creation of the cybrid model. Mitochondrial DNA (mtDNA) isolation from donors with subsequent transfer to immortalized cells depleted of endogenous mtDNA creates cytoplasmic hybrid (cybrid) cell lines that recapitulate at cellular and sub-cellular levels any specific functional abnormalities characteristic of the transferred mtDNA. The cybrid technique is useful both in demonstrating the functional consequences of known mtDNA mutations (Chomyn et al., 1994) and for screening for mtDNA mutations in candidate diseases (Swerdlow and Hanna, 1996; Swerdlow et al., 1996). Cybrid models, in which mtDNA from platelets of living AD patients is expressed in replicating human neural cells initially devoid of their own endogenous mtDNA (rho(0) cells) revealed that decreased cytochrome oxidase activity, increased oxidative stress, increased Aβ production, activation of detrimental intracellular signaling and caspases, accelerated mtDNA proliferation, and abnormal mitochondrial morphology and transport can be transmitted through expression of mtDNA from living AD patients (Onyango et al., 2006).

Many studies have applied to cells synthetic Aβ peptides to create *in vitro* models. Many recent studies have used oligomers of human Aβ prepared from synthetic Aβ peptides (Wang et al., 2004), isolated from transfected cell lines (Walsh and Selkoe, 2007) or purified from brains affected by AD (Shankar et al., 2008). However, this AD *in vitro* models show some limitations, because the research groups reported different results. This divergence could depend on the form of Aβ because different Aβ assemblies or conformation could have diverse targets.

### COMPUTATIONAL MODELS

Another way to study a complex disease like AD is to model the pathology with the assistance of bioinformatic. In physics a complex system is a system in which the individual parts are affected by local interactions, short range of action, which cause changes in the overall structure. Science can detect local changes, but cannot predict a future state of the system considered as a whole. The complexity of brain dynamics may be too high for us to conceptually understand the brain’s function in detail (Duch, 2007); computational model may collect a number of essential features of brain dynamics leading to a model that behaves like whole brain interactions and processes. Initially cognitive functions were considered as a sequence of processing stage; perceptual processes are followed by attentional processes that transfer information to short-term memory and thus to long-term memory (Atkinson and Shiffrin, 1968), and these are often represented as a series of box-and-arrow diagrams. From this point of view, brain dysfunctions are treated as disturbance in the information flow. In 1986 was published a “*PDP book*” (Parallel Distributed Processing: Exploration in the Microstructure of Cognition), now known as “connectionism,” in which authors focus the attention on the general proprieties of parallel information processing, referring to the fact that a lot of different information can be processed simultaneously, drawing analogies with human information processing (McClelland and Rumelhart, 1986; McClelland et al., 1995). The computational model based on this concept is named *neural network;* this model is composed of array of simple information-carrying units called nodes that respond to a particular set of *inputs* and produce a restricted set of *outputs.* The responsiveness of a node depends on how strongly it is connected to other nodes in the network and how active the other nodes are. A network of nodes interacting with each other through connections of different strengths, and each network node, called *an artificial neuron*, may represent a single neuron of a specific type, a generic neuron, a group of few neurons, a neural assembly, or an elementary function realized in an unspecified way by some brain area (Duch, 2007). Since AD is characterized by loss of memory, neural model may represent a powerful tool to unravel the relation between changes at cellular level and various clinical manifestations of the disease (McClelland et al., 1995; Hasselmo and McClelland, 1999).

#### The synaptic deletion and compensation model

The synaptic deletion and compensation model was conceived initially by Horn and subsequently developed by Ruppin (Horn et al., 1993; Ruppin and Reggia, 1995). The models of artificial neural progression of AD depart from the concept that cell death in AD is generally limited within 10% of the neuronal population, value that does not reflect the level of cognitive impairment of the patients: the primary factor responsible for cognitive deficits in AD is the loss of approximately 50% of synaptic connections rather than the mortality of neurons. Brain tries to compensate the loss of these connections by strengthening the remaining; this mechanism is successful in the early stages of development of AD by limiting the cognitive deficits but not more in the terminal phase: understanding how these two processes can influence memory, and what the best compensation strategies that may slow down the deterioration process are, represents the main questions of this model. This model has demonstrated that the loss of synaptic connections gives rise to memory loss and distortion of the learned pattern, even if the rate of memory deterioration can be reduced by increasing the weight of the remaining connections for a constant multiplication factor (Horn et al., 1996). Depending on the compensation strategy after the same evolution period various degrees of deterioration are obtained; this may explain why patients with similar density of synaptic connections per unit of cortical volume show quite different cognitive impairments.

#### The synaptic runaway model

This model was developed by Hasselmo (1991, 1997) and is focused on a different phenomenon: in models of associative memory, storing a memory as a pattern of neuronal spatiotemporal activation is associated with the storage of other connected memories. The storage of a new memory activates analogs pattern that can interfere with earlier associations if there was an overlap between patterns or if the memory capacity is exhausted. This interference results in a significant increase in the number of associations stored by the network that can also lead to a pathological increase of the strength of synaptic connections, which gives rise to an increase of neuronal activity, high metabolic demand, and eventually cells death, phenomenon known as excitotoxicity. The model has also been used to show that in the brain there are two separate mechanisms of memory, one for encoding and the other for the recall, in order to minimize the excitotoxicity induced interference. According to this hypothesis, the inhibitory and excitatory cholinergic neuromodulation is the main factor controlling the switching from one to another mode. Under normal conditions the neuromodulation is sufficient to prevent changes in runaway synaptic modification (RSM), while in pathological conditions such AD the RSM is inevitable. Even healthy subjects may run into RSM due to overloading of memory. However, it has been shown that the threshold levels for the initiation of RSM in AD are much lower compared to controls (Siegle and Hasselmo, 2002).

## CONCLUSION

Overall, these models have played a major role in defining critical disease-related mechanisms and in exploring novel potential therapeutic approaches. The purpose of neuroscientist is to obtain helpful experimental model of human brain disorders (Torres-Aleman, 2008), able to give the opportunity to reach significant advancements in the understanding of pathological pathways, to characterize and study the molecular and biochemical mechanisms that are impossible to study in human samples. Current AD models rely on information gathered from inherited forms of the disease, far less common than sporadic ones but indistinguishable from the clinical point of view. Thus, both early FAD and LOAD forms progressively develop cognitive deterioration that in early stages affect memory systems and at late phases impair all cognitive spheres of daily life activities (Albert et al., 1996).

We have described as invertebrate model systems have many practical advantages for modeling human disease: short generation time, high fertility, readily available techniques for genetic manipulation, ease of feeding therapeutics, and low cost of maintenance. These advantages make them amenable for large-scale phenotype-based genetic screens and pharmacological manipulations. Thus, they have been proven excellent systems for studying the normal function of human genes and pathways linked to neurodegenerative diseases such as AD and other tauopathies. In the future, forward genetic screens using these models can help us in better understanding the putative genes and pathways associated with AD. When pairing the information we can gather from these “simple” invertebrate models systems with their vertebrate counterparts such as mice and rats, an enhanced determination of which specific experimental approaches to be taken, or future directions for the field can be achieved. Ultimately, this will be translated into the discovery of novel gene targets and novel diagnostic and therapeutic strategies against AD and other dementias.

As vertebrate model we highlight the main advances that zebrafish offers in order to understand the pathological mechanisms of AD. This vertebrate is an interesting tool that can be strategically incorporated into the analysis of the neurodegeneration cascade, covering the existing gap between the drug discovery in cellular models and the preclinical assays in rodents. In any case, zebrafish should be used in large scale before pharmacological validation in rodent models, especially because of its larva–adult duality. Although we have provided diverse examples to demonstrate the scope for zebrafish to model AD, several pieces of the puzzle are still lacking. For example, a better understanding of the comparative brain anatomy and physiology of adult zebrafish will be required, and more transgenic zebrafish to model an AD-like pathology are a current need. So a more effort must be done to definitely optimize the zebrafish as a valid model for AD.

As in human, transgenic mice show an overexpression of Aβ and development of senile plaques; the main observation arising from mouse model was that an excess of Aβ is enough to induce cognitive impairment and synaptic dysfunction even in the absence of NTFs or neuronal loss (Gotz et al., 2004), confirming that Aβ overproduction must trigger AD, before tau deposition. This finding substantiated the amyloid cascade hypothesis of AD. According to this hypothesis, accumulation of Aβ in the brain is the primary influence driving the disease pathogenesis (Hardy and Selkoe, 2002). The rest of the disease process, including formation of neurofibrillary tangles, is proposed to result from an imbalance between Aβ production and Aβ clearance. Some of the strongest data supporting tau pathology as a downstream event of Aβ accumulation have come from studies in 3xTg mice. In these mice, the appearance of intraneuronal Aβ precedes somatodendritic accumulation of tau (Oddo et al., 2004). Furthermore, removal of intraneuronal Aβ via immunotherapy leads to the removal of somatodendritic tau shortly afterward (Oddo et al., 2004). Transgenic mice have also allowed us to study aspects of the disease that are inaccessible in humans, as the correlation between synaptic deficits and disease’s progression. In accordance with amyloid hypothesis of AD (Hardy and Selkoe, 2002) the species responsible for synaptic and cognitive dysfunction are soluble Aβ oligomers, and this can explain the lack of correlation between the number of Aβ plaques and the degree of dementia in humans and the heavy correlation with low-molecular-weight soluble Aβ species (Funato et al., 1999; McLean et al., 1999; Klein et al., 2001). Several groups conducted deep cognitive investigation in transgenic mice and the results are in line with this finding: mice at different age do not display any correlation between the number of senile plaques and memory dysfunction, moreover cognitive deficits precede plaque deposition (Westerman et al., 2002; Van Dam and De Deyn, 2006; Balducci et al., 2010).

Due to the complexity of the disease, no transgenic line is able to recapitulate all aspects of the pathology, suggesting probably the limitation of using a rodent system to reproduce a human disease process that take several decades to develop and which primarily involves higher cognitive function. Many factors may affect the ability of transgenic mice to model human disease. These include obvious anatomical and morphological differences as well as the possibly difference in critical protein such as tau or APP (Gama Sosa et al., 2012). The main question arising from this evidence is whether transgenic mice do really mirror the temporal profile of the neuropathological process of human AD. All age-related neurodegenerative disease develop in context of aging, that is believed to be the results of the gradual loss metabolic and genetic assets necessary to preserve the integrity and functionality of all cellular components (Ljubuncic and Reznick, 2009); furthermore non-physiological 5–10-fold overexpression of human APP in transgenic mice could accelerate the pathological role of the overexpressed protein, while in human an increase of 50% is enough for amyloid plaques deposition (Duff and Suleman, 2004). Another important difference between mice and AD patients is the structure of the Aβ deposition; mice show a compact core plaques while in human the core density is lower and the morphology amorphous (Roher and Kokjohn, 2002). Regarding soluble Aβ oligomers, biochemical analysis indicated that there are difference not only between humans and mice, but also among transgenic lines; in this frame, it is interesting to point out again that different genetic strains may produce different amounts of Aβ, forming potentially different oligomeric forms and in different genetic background.

In light of these considerations, we should take into consideration non-transgenic AD mice models, For instance, interfering with synaptic ADAM10 localization/activity for 2 weeks affects APP metabolism, increasing APP amyloidogenic cleavage with a modest but significant raise of Aβ release and of low-n Aβ aggregate production (Epis et al., 2010). This subtle increase in Aβ appears particularly promising when compared with data collected from studies in engineered animal models, where accumulation of soluble Aβ is normally reported in older animals, i.e., 9–13 months of age (Holcomb et al., 1998; Oddo et al., 2003a). Similarly, even in triple transgenic mice, Aβ oligomers can be revealed after 13 months of age (Oddo et al., 2003b, 2006). Notably, in this non-transgenic model, the slight increase of both soluble Aβ and its low-n aggregates is evident after just 2 weeks of treatment, thus it appears as a promising model to study early phases of AD.

New perspectives are also emerging to deal with the difficulties of modeling AD pathology.

A growing amount of evidence suggests that stem cell-based therapies could prove beneficial in AD, albeit via indirect mechanisms rather than cellular replacement. Studies of embryonic, neural, and iPSCs are also beginning to unravel the normal and pathogenic function of AD-associated genes and may provide powerful new approaches to model this disorder. Future work will hopefully clarify the potential of stem cells to treat AD and decipher the complex genetic differences that predispose a person to developing this devastating disease.

We have also described two computational models that can be seen as complementary rather than competitive. The AD may develop through different routes: on the one hand the loss of synapses and an insufficient compensation lead to small brain damage, the other the synaptic runaway may lead to death of hyperactive neurons with significant structural damage of the brain. The assumption that computational model reflects real neural mechanism leads to several therapeutics suggestions: they may help to slow down the degeneration of synaptic connections and thus the development of the disease in its early stage (Duch, 2000, 2007). Furthermore, neural model provide a new level of reasoning about brain disease level that cannot be adequately described in the language of psychiatry or psychopharmacology (Duch, 1997).

In conclusion the development of adequate animal models mimicking all stages of AD progression and merging convergent pathways of pathogenesis still represents a need for research on AD. However, the complementary use of several models, with distinct pros and contras, will help to understand the molecular basis of the AD, and to develop novel strategies for AD prevention and therapy.

## Conflict of Interest Statement

The authors declare that the research was conducted in the absence of any commercial or financial relationships that could be construed as a potential conflict of interest.
